# Patients' experiences of engaging with electronic Patient Reported Outcome Measures (PROMs) after the completion of radiation therapy for breast cancer: a pilot service evaluation

**DOI:** 10.1002/jmrs.711

**Published:** 2023-08-07

**Authors:** Jane Hughes, Terri Flood

**Affiliations:** ^1^ North West Cancer Centre, Altnagelvin Hospital Western Health and Social Care Trust Derry UK; ^2^ Ulster University Londonderry UK

**Keywords:** breast, experience, follow-up, patient, PROM, review, radiation therapy

## Abstract

**Introduction:**

Over 60 % of people who develop breast cancer will receive radiation therapy (RT) as part of their treatment. Side effects of RT may include inflammation, erythema, desquamation and fatigue. Electronic Patient Reported Outcomes Measures (ePROMs) enable patients to report side effects prior to their scheduled post‐RT appointment. This pilot service evaluation aims to explore patients' perceptions regarding the value of the ePROM system, ease of its use and barriers to using the system, after breast irradiation.

**Methods:**

From July–November 2021, evaluation surveys were posted to 100 people who had received RT to their breast to explore their experience of using the ePROM. Ethical approval was obtained through Ulster University and the Western Health and Social Care Trust (WHSCT), Northern Ireland.

**Results:**

Fifty‐two people responded to the survey, of which 27 respondents indicated that they had accessed the ePROM. Despite few participants experiencing significant side effects, the majority of participants recommended the ePROM indicating that it was an important source of support. Those who experienced significant side effects found the system to be prompt and effective. Barriers to accessing the ePROM included technical issues with the link, concerns about confidentiality and forgetting to access the link. Access to the ePROM increased with higher education levels.

**Conclusions:**

This pilot service evaluation demonstrated that ePROMs are valued by patients and can provide rapid real‐time access to support, offering individual care and reassurance. For patients with longer RT schedules (>10 fractions), the introduction of ePROMs during RT was viewed favourably by participants. All patients may benefit from the option of receiving ePROMs post‐RT.

## Graphical abstract

A pilot service evaluation exploring patients' perceptions regarding the value of an Electronic Patient Reported Outcomes Measures system, ease of its use and barriers to using the system, after radiation therapy for breast cancer.

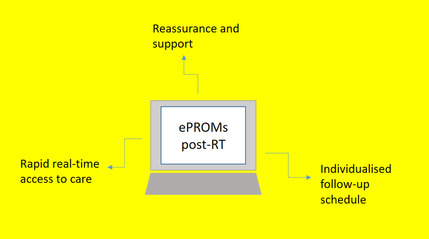

## Introduction

Approximately 63% of patients diagnosed with breast cancer will receive radiation therapy (RT) as part of their primary cancer treatment.^1^ Over the last decade, 40 Gy in 15 fractions became the standard dose and fractionation for RT to the breast in early stage breast cancer.^2^, ^3^ However, in March–April 2020, the Institute of Cancer Research (ICR) recommended the use of 26 Gy in five fractions for all adjuvant treatment of early breast cancer based on findings from the Fast Forward trial.^2^, ^4^ As nodal irradiation was not included in this trial, 40 Gy in 15 fractions remains the recommended standard for patients within this group.^2^ Selected patients may also receive a ‘boost’ to the initial tumour site resulting in up to five additional treatments to these standard schedules.^2^ While RT techniques have improved dramatically over the last decade, patients still experience localised side effects as a result of irradiating surrounding healthy tissue.^5^ Early side‐effects of breast irradiation can appear within days to weeks and include inflammation, erythema and desquamation of the skin.^3^ As most breast irradiation is now delivered over 5 days, early side effects are less likely to be managed during RT treatment delivery. Throughout the United Kingdom (UK), post‐RT follow‐up schedules for assessment of side effects, are set by individual departments based on recommendations from the Royal College of Radiologists.^6^, ^7^, ^8^, ^9^ In the North West Cancer Centre (NWCC), one of two cancer centres in Northern Ireland, patients who receive breast irradiation are routinely reviewed 12 weeks post‐RT. However, an audit conducted by NWCC in 2019, found that when these patients were reviewed 12 weeks post‐RT, some patients reported higher‐grade toxicities compared to those recorded during their final week of treatment. Despite this finding, the audit revealed that contact from patients prior to their 12‐week appointment was rare, with patients mostly waiting until their appointment to discuss concerns. Hence, it was concluded that it may be necessary to assess some patients after RT for breast cancer, prior to the 12‐week follow‐up appointment, but without reverting to shorter follow‐up times which is resource intensive and unnecessary for many patients.^10^

To address this concern, NWCC explored the possibility of implementing electronic Patient Reported Outcome Measures (PROMs). PROMs involve the collection of information directly from patients regarding their medical condition, side effects of treatment and other health‐related constructs.^11^, ^12^, ^13^ Numerous studies support the introduction of PROMs and suggest that they can increase communication between patients and clinicians leading to better detection of problems^14^ and improved understanding of side effects.^15^, ^16^ Collated findings by the National Institute for Health and Care Excellence (NICE) indicate that patients want to be active participants in their own healthcare^17^ and the introduction of PROMs provides an outlet to support self‐directed care. Additionally, emerging models for healthcare, based on engagement and involvement, all champion proactive, patient self‐management.^18^, ^19^, ^20^

Although PROMs are a useful tool when combined with routine practice, there are few studies that have explored PROMs as a standalone method of self‐directed care.^21^ In addition, the majority of studies focus on the use of PROMs while the patient is receiving treatment, with few exploring its value for post‐treatment care.^15^, ^16^ Studies which have explored PROM use for post‐treatment care^15^, ^16^ have not specifically looked at radiotherapy side‐effects post‐treatment and the timing of the PROM completion is unclear. Additionally, these studies required the patients to complete the PROM in the clinical setting and therefore have not tested the success of the ePROM when completed remotely.^15^, ^16^ Subsequently, additional research is needed to obtain the patient perspective regarding the use of remote ePROMs for self‐directed care post‐RT.^15^

To address this gap in post‐treatment care, an electronic PROM (ePROM) system, Penguin (Cievert Ltd, Gateshead, UK) was introduced into NWCC for digital self‐directed care, to encourage reporting of acute toxicities in real time, in alignment with the *NHS Long‐Term Plan* for access to digitised care.^22^ Due to the high number of patients who receive breast irradiation compared to other sites, this group of patients was selected for a pilot service evaluation of the system. The primary aim of this evaluation was to establish patients' perspectives regarding the value of the ePROM system, ease of use of the system and potential barriers to using the system, post‐RT.

## Methods

### Study design

This pilot service evaluation involved a single cancer centre, NWCC, and utilised an anonymous postal evaluation survey to capture data regarding patients' experiences of accessing and using the ePROM. A survey was chosen as the information‐gathering method, as it allows for data to be collected from a large, targeted population.^23^ Ethical approval for this evaluation was obtained through the Nursing and Health Research Ethics Filter Committee at Ulster University, as well as the Western Health and Social Care Trust (WHSCT).

Penguin, the ePROM software, was supplied as part of an ongoing departmental contract with Cievert at no additional charge and in line with local change control processes.

### Participant recruitment

Participants were eligible to be enrolled into the study if they were a minimum age of 18 years old and completed radical RT for breast cancer in NWCC between July 2021 and November 2021. Radical RT treatment is defined as treatment given to cure or improve survival substantially.^24^ All radical breast cancer patients who consented to RT from July 2021 were offered the opportunity to receive ePROM communications via e‐mail to enable them to self‐report side effects electronically after completion of RT. This information was provided to them by the lead author, who is also the radiation therapist primarily involved in the implementation of this system. Information regarding the ePROM was provided to them in their last week of treatment, where they also received information regarding the pilot service evaluation survey, at this time. Patients who expressed that they would like to use the ePROM post‐RT were entered into Penguin, which generates an electronic questionnaire when their diagnosis is added. The ePROM was manually scheduled to be sent once per week between week 6 and week 12 after their last RT treatment. To access the link to the ePROM, participants were required to enter their postal code and date of birth. All participants were supplied with end‐of‐treatment literature and continued to have a face‐to‐face or telephone appointments scheduled at approximately 12 weeks post‐RT (depending on their clinician's availability). The ePROM used a scoring system adapted from the Radiation Therapy Oncology Group (RTOG) and Common Terminology Criteria for Adverse Effects (CTCAE) grading system,^25^ to improve patient comprehension. This adapted tool was already widely used within the oncology department for reporting of side‐effects. For each question, clinicians agreed on threshold grades for each side‐effect, beyond which responses were flagged for their attention (see Supporting Information, Appendix S1). If the participant selected these grades, an email alert was automatically sent to the designated team. The email was re‐sent regularly to clinicians until the flag was actioned. Participants who returned their ePROM without significant issues did not need any intervention until their scheduled 12‐week follow‐up appointment. Participants could choose to complete as many of the ePROMs as they wished between week 6 and week 12 post‐RT; there was no minimum number of completions required. Participants did not complete any ePROMs during their RT in this study.

### Participant evaluation postal survey

Once the participants' 12‐week RT follow‐up date had passed, a Participant Information Sheet (PIS), including the evaluation survey (see Supporting Information, Appendix S2) was supplied with a stamped addressed envelope to elicit feedback regarding their experience of using the ePROM prior to their 12‐week appointment.

### Sample and sampling

Approximately 1450 women in Northern Ireland are diagnosed with breast cancer every year.^26^ As approximately 63% of these patients will be offered RT,^1^ then approximately 914 women would be eligible for the study over a period of 1 year. As this pilot service evaluation was conducted over a period of less than 6 months and at least half of all patients with breast cancer would be treated in the other Northern Ireland cancer centre (Belfast City Hospital), then approximately 229 women may have been eligible to participate.

It is generally accepted that pilot studies should be large enough to provide useful information but do not need to adhere to the narrow margins of error and confidence levels associated with full studies.^27^ Consequently, the researchers aligned to Schober et al.'s^28^ methodology, aiming for a margin of error of 10–15% on a 95% confidence level. Using the Raosoft sample size calculator,^29^ it was, therefore, determined that 37–68 participants would need to be successfully recruited.

Based on an anticipated response rate of approximately 50%,^30^ the first 100 participants who had access to the weekly ePROM, were sent an anonymous survey.

### Data analysis

*Descriptive statistics* were used to analyse data for single categorical variables and included frequencies and percentages. Inferential statistics were used to explore whether participant demographic variables influenced participants' ability to access the ePROM. Age and educational level were assessed for normality using the Shapiro–Wilk statistic and visualisation of the graphical data^31^, ^32^ and assessed using the Mann–Whitney U test or independent t‐test.^33^ Employment status and residential location were assessed using the chi‐square test.^30^, ^34^ An alpha value of 0.05 was set for statistical significance for all tests.

Qualitative data were analysed using thematic analysis where open, axial and selective coding was applied to the data as described by Williams & Moser.^35^ In the open coding phase, words and phrases were assimilated iteratively into codes by one researcher for each question with a qualitative component. The second researcher independently completed this first step strengthening the rigour of the data analysis.^36^ Open codes were then discussed and refined between the two researchers through reflection and reflexivity in a non‐linear process until agreement of codes was reached, further increasing the rigour of the data analysis.^36^ Axial and selective coding were performed as a collaborative task between the two researchers where emergent themes were discussed and established. Due to the low volume of qualitative data, this process was relatively uncomplicated compared to the complexity inherent in interviews or focus groups.

## Results

Of the 100 participants who were sent a feedback survey, 52 participants returned their completed survey detailing their experience of using the ePROM.

### Section A

#### Demographic information

Participants most frequently indicated that they were employed but on leave (*n* = 19; 36.5%). Twenty‐four participants (46.2%) indicated having an education level higher than secondary school. Participants most frequently reported being between 46 and 55 years old (*n* = 18; 34.6%) with 21 participants (40.4%) indicating that they were older than 55 years old. An equal number of participants reported living in either the town or countryside (*n* = 19; 36.5%).

Participants most frequently indicated that they had received 10 fractions of RT (*n* = 19; 36.5%), followed closely by 5 fractions (*n* = 18; 34.6%). Fifteen participants (28.8%) indicated that they received ≥15 fractions of RT. All participants identified that they had surgery and a further 25 participants (48.1%) identified that they received chemotherapy in addition to surgery. Four participants (7.7%) indicated that they required wound dressing and four participants (7.7%) indicated that they required emotional support during RT. Nine participants (17.3%) identified that they required physiotherapy for complications related to their cancer, during RT treatment. See Table 1 for full demographic details.

**Table 1:** Demographics of participants.

Variable	*n*	(%)
Employment Status		
On leave from work	19	36.5
Working part‐time	7	13.5
Working full‐time	10	19.2
Retired	9	17.3
Other	4	7.7
No response	3	5.8
Educational level		
Postgraduate degree	7	13.5
Undergraduate degree	9	17.3
Diploma	8	15.4
Secondary	20	38.5
Primary	1	1.9
Other	4	7.7
No response	3	5.8
Age range		
18–25 years	0	0
26–35 years	2	3.8
36–45 years	8	15.4
46–55 years	18	34.6
56–65 years	12	23.1
66–75 years	7	13.5
76–85 years	2	3.8
+85 years	0	0
No response	3	5.8
Place of residence		
City	5	9.6
Town	19	36.5
Village	4	7.7
Countryside	19	36.5
Remote	2	3.8
No response	3	5.8
Number of radiotherapy treatments		
5	18	34.6
10	19	36.5
15	6	11.5
20	8	15.4
25	0	0
30	0	0
33	1	1.9
Neo‐adjuvant treatment		
Surgery	52	100.0
Chemotherapy	25	48.1
Hormone blocking therapy	14	26.9
Other	0	0
Supportive Interventions during radiotherapy		
Wound dressing	4	7.7
Seroma drainage	0	0
Emotional support	4	7.7
Other	2	3.8
Visits with Allied Health Professionals during radiotherapy		
Physiotherapist	9	17.3
Speech & Language Therapist	0	0
Occupational Therapist	0	0
Dietician	0	0
Hospital‐based social worker	0	0
Other	1	1.9

#### Access to and completion of the ePROM

Twenty‐seven participants (51.9%) identified that they were able to access the ePROM. Reasons reported for not accessing the ePROM included issues with the link, issues with access and not receiving the email. Some comments included:

Could not access this because it told me each time that it had expired” (P21) “Unfortunately the email was sent as a spam and I could not access the email. I tried several things but still could not open it. (P42)

Wouldn't accept code. (P1)

Twenty‐four participants (46.2%) identified that they completed an ePROM. Common reasons reported for not completing an ePROM included poor internet connection and participant's own forgetfulness. Comments included:

Poor internet. (P13)

Completely slipped my mind. (P16)

Five participants (9.6%) identified that their ePROM required a response from the RT team. See Table 2.

**Table 2:** Participants' access to and use of the ePROM.

	Yes	No	No response	Total
	*n* (%)	*n* (%)	*n* (%)	*n* (%)
Were you able to access the ePROM questionnaire?	27 (51.9)	24 (46.2)	1 (1.9)	52 (100)
Did you complete the ePROM questionnaire?	24 (46.2)	16 (30.8)	12 (23.1)	52 (100)
Did you use the ePROM to report that you were experiencing side‐effects that required a response?	5 (9.6)	47 (90.4)	0	52 (100)

Four of the five participants who required a response from the RT team, identified that they were contacted by a member of the team; the remaining one participant did not respond to this question. These four participants indicated that they were contacted within 1–3 days of submission of the ePROM. Three of the four participants who required a response, commented that the quality of care that they subsequently received was adequate, good and very good respectively; the other 2 participants did not complete this question. See Table 3 for full details.

**Table 3:** Participants' report on the response that they received from the radiotherapy team through engagement with the ePROM system.

		Yes		No		No response	N/A	Total
		*n* (%)		*n* (%)		*n* (%)	*n* (%)	*n* (%)
If you reported side‐effects which required a response, were you then contacted by a member of the radiotherapy clinical team?		4 (7.7)		0 (0)		1 (1.9)	47 (90.4)	52 (100)
	**1 day** ***n* (%)**	**2 days** ***n* (%)**		**3 days** ***n* (%)**		**No response** ***n* (%)**	**N/A** ***n* (%)**	**Total** ***n* (%)**
If contacted, how long did it take to get a response?	2 (3.8)	1 (1.9)		1 (1.9)		1 (1.9)	47 (90.4)	52 (100)
	**Very poor** ***n* (%)**	**Poor** ***n* (%)**	**Adequate** ***n* (%)**	**Good** ***n* (%)**	**Very good** ***n* (%)**	**No response** ***n* (%)**	**N/A** ***n* (%)**	**Total** ***n* (%)**
Overall, how would you rate the quality of care that you received through completion of the ePROM?	0 (0)	0 (0)	1 (1.9)	1 (1.9)	1 (1.9)	2 (3.8)	47 (90.4)	52 (100)
		**Yes** ***n* (%)**		**No** ***n* (%)**		**No response** ***n* (%)**	**N/A** ***n* (%)**	**Total** ***n* (%)**
At your 12 week follow up appointment, the clinician will have reviewed your ePROM questionnaire submission from 6 weeks after your Radiotherapy finished. Did you feel that this aided the review process?		15 (28.8)		4 (7.7)		15 (28.8)	18 (34.6)	52 (100)

Fifteen participants (28.8%) identified that they felt that the ePROM aided the review process, with comments such as:

It put my mind to rest. (P6)

It informed the questions asked in the telephone follow up appointment. (P26)

Yes because she already had knowledge of my progress. (P35)

Other participants' comments indicated their uncertainty regarding the value of the ePROM. Three of the four participants that answered no, commented:

Not sure that this happened. (P10)

My response to the ePROM wasn't specifically mentioned at my 12 week review. (P12)

Not mentioned. (P30)

### Section B

The majority of participants (*n* = 36; 69.2%) identified that they felt the ePROM was sufficiently explained, with only three participants (5.8%) reporting that it was not sufficiently explained. Of the three participants who felt that the ePROM was not sufficiently explained, two participants felt this was due to their own memory of the information session. See Table 4. Comments from the latter included:

**Table 4:** Participants' perceptions of information received regarding the ePROM and the value of the ePROM.

	Yes	No	No response	Other	Total
	*n* (%)	*n* (%)	*n* (%)	*n* (%)	*n* (%)
Did you feel that the ePROM system was explained sufficiently?	36 (69.2)	3 (5.8)	13 (25)	0 (0)	52 (100)
Were you apprehensive to use the ePROM system?	6 (11.5)	30 (57.7)	12 (23.1)	4 (7.7)	52 (100)
Would you recommend using the ePROM system to report side effects after your radiotherapy treatment has finished?	32 (61.5)	4 (7.7)	13 (25)	3 (5.8)	52 (100)
Do you think that ePROM questionnaires should be sent whilst receiving radiotherapy to report side effects?	31 (59.6)	6 (11.5)	12 (23.1)	3 (5.8)	52 (100)
Do you feel the information and support you received has helped you to manage your post‐radiotherapy care?	36 (69.2)	3 (5.8)	10 (19.2)	3 (5.8)	52 (100)

If it was explained, I don't remember. (P41)

… because I was stressed, I did not understand what I was being told or forgot it… (P6)

Participants most frequently (*n* = 30; 57.7%) indicated that they were not apprehensive about using the ePROM system, with only six participants (11.5%) reporting apprehension. Comments from the latter included:

I thought it was a scam at first, but then emailed the WHSCT and found out that it was legitimate. (P6)

Confidentiality. (P14)

The majority of participants (*n* = 32; 61.5%) identified that they would recommend using an ePROM to report side effects post‐RT. See Table 4 for full details.

One participant commented:

If rolled out I would say it would allow clinicians to deal with issues quicker and time efficient. (P40)

Only four participants (7.7%) reported that they would not recommend it, with comments including:

Prefer speaking to a professional directly. (P4)

I would prefer to talk to a clinical on the phone or in person. (P23)

Two of the four participants commented that they would not recommend it because the link did not work (P28, P37).

The majority of participants (*n* = 31; 59.6%) agreed that the ePROM should also be sent out while they were receiving RT treatment. Of the 15 participants (28.8%) who received ≥15 RT treatments, 12 of these 15 participants agreed with this statement with the remaining three participants not answering this question.

The six participants (11.5%) who did not think this should occur all received either five or 10 RT treatments. Two of the six participants commented that this was because they had no side effects during the treatment. Comments included:

because you need to finish the treatment and see what the following week is like. (P21)

My side effects arrived 2 weeks later. (P19)

The majority of participants (*n* = 36; 69.2%) indicated that they felt that they received enough information and support to help manage post‐RT care. Comments included:

I was given a checklist to monitor my health during and after radiotherapy that was very helpful. (P12)

The information and support I received prepared me for the side effects and timeline associated with them, especially in the first month after radiotherapy. (P26)

Of the three participants (5.8%) who disagreed, only one comment was provided:

No to be honest they were friendly every day during treatment but when it's over you're on your own. (P29)

Eleven participants (21.1%) added additional comments, a cross‐section of which is shown in Table 5.

**Table 5:** Comments from participants regarding use of the ePROM.

“Maybe a little clearer who the emails are from or maybe write out the email address so that we recognise who it is from exactly. Being a traumatic time we don’t always hear or remember what is said.” (P6)
“I wasn't clear on whether I should complete the form multiple times (this may have been said to me, I can’t recall). I had no major issues so I didn’t feel the need to report my progress when I felt well from an early stage.” (P12)
“For serious and painful side effects I would probably prefer telephone/ face to face consultation but I did not experience these and was prepared and equipped to deal with pinkish hue, heat, slight itchiness explained during face to face consultation” (P26)
“I think if rolled out it would be very beneficial as not all patients are the same and their treatment may incur questions that are not the “norm” so if clinician aware then it does save time.” (P40)

### Relationship between participant demographics (Section A) and access to the ePROM (Section B)

Out of the 52 participants who completed the evaluation survey, one participant did not indicate whether they assessed the ePROM and therefore the data analysis in this section is based on the responses of 51 participants.

To assess if education level had an impact on whether participants accessed the ePROMs, education level was allocated a score of 1 to 5 for increasing participant education level from completion of primary school (score of 1) to receipt of a postgraduate degree (score of 5). Seven participants either did not indicate their level of education or chose ‘other’ so were removed from the data analysis. Therefore 44 participants were included in this analysis. The researchers explored the alternative hypothesis which was that those with higher education levels would access the ePROM more frequently.

To assess if age had an impact on whether participants accessed the ePROM, age was also allocated a score of 1 to 8 with the lowest age range being allocated a score of 1 and the highest age range being allocated a score of 8. Three participants did not complete this question resulting in 48 participants being included in the data analysis. The alternative hypothesis proposed that accessing the ePROM would increase with decreasing age.

Due to the visualisation of the graphical data and a Shapiro–Wilk statistic of <0.05 in both data sets, the data was considered not to be normally distributed for educational level or age. Consequently, a one‐tailed non‐parametric Mann–Whitney *U* test was utilised to analyse this data. This test demonstrated that there was a significant difference (Mean rank score 18.68 vs. 25.69; *U* = 163.5, *P* = 0.029) between the education level of participants who accessed the ePROM compared to those who did not access the ePROM; those who accessed the ePROM having higher levels of education. A one‐tailed independent *t*‐test also confirmed the finding with a *P*‐value of 0.018 (*t* = −2.253; df = 41.92) and mean scores of 2.65 versus 3.38 for those who did not access the ePROM and who did access the ePROM respectively. Therefore the alternative hypothesis was accepted; access to the ePROM increases with increasing educational level.

Using a one‐tailed non‐parametric Mann–Whitney *U* test, no significant difference was found in age between those who accessed the ePROM and those who did not (*U* = 286, *P* = 0.974); the mean rank scores being just over 24 in both groups. Therefore, the null hypothesis was accepted and it was concluded that there was no difference between the groups.

Due to the low number of participants who indicated that they lived in the city or in rural areas, the participants were further categorised as either living in highly populated areas (city or town: *n* = 24) or lower populated areas (village, countryside or rural: *n* = 24). Three participants did not indicate where they lived resulting in analysis based on 48 participant responses. A 2 × 2 matrix Chi‐square test was used to assess the relationship between where participants lived and whether this impacted their access to the ePROM. This test indicated that there was no significant association between accessing the ePROM and residential location X^2^ (1, *n* = 48) = 0.751, *P* = 0.386.

Similarly, employment status was re‐categorised into 2 categories; employed (*n* = 26) and other (including retired) (*n* = 13). A 2 × 2 matrix Chi square test was used to access the relationship between employment status and whether this impacted participant access to the ePROM. This test indicated that there was no association between accessing the ePROM and employment status X^2^ (1, *n* = 48) = 1.326, *P* = 0.25).

A statistician was consulted to confirm the findings in this section.

## Discussion

### Participants' perspectives regarding the value of the ePROM system

The majority of participants who contributed to this pilot service evaluation, recommended that the ePROM should be integrated into post‐RT care for reporting of side‐effects. They indicated that the ePROM assisted them in rapidly reporting arising problems and felt reassured by its ability to accurately inform their RT team of their side‐effects prior to their 12 week post‐RT appointment. While the numbers of participants who reported significant issues were small, the majority of these participants expressed satisfaction with how they were contacted in a timely manner and had their issues resolved. This evaluation suggests that weekly ePROMs post‐RT may allow for follow‐up appointments to be more widely dispersed, reducing unnecessary patient travel and in turn allowing for more efficient allocation of resources, as well as ensuring patient centred care; all of which are a priority for health care providers.^18^, ^19^, ^20^ The introduction of ePROMs for self‐directed aftercare would also mean that patients can report toxicities before their follow‐up appointment resulting in faster triage and unnecessary escalation of toxicities.^15^, ^16^, ^37^, ^38^ On a larger scale, the data gathered can be used to establish anticipated side‐effects and screen for recurrence.

**Figure 1:**
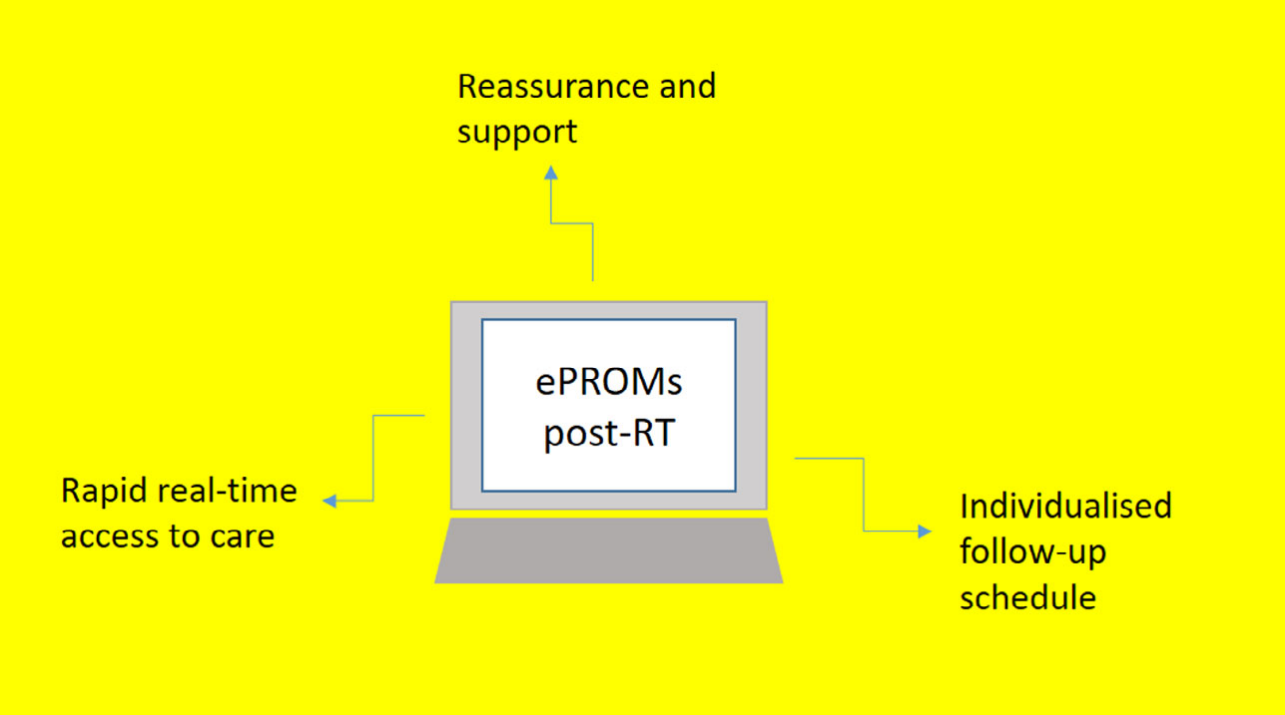
Summary of perceived benefits of ePROMs post‐RT.

Participants described feeling supported by their RT team through the ePROM weekly communication even where side‐effects were mild. Given that approximately 30% of patients diagnosed with breast cancer experience significant fear and anxiety prior to RT,^39^ the ePROM may act as an important source of support to reassure patients while waiting for their post‐RT appointment. It is worth noting that the ePROM in this study did not assess distress or anxiety. However, other studies have reported improved management of anxiety and depression through the integration of validated scales like the Hospital and Depression Scale (HADS) within the ePROM.^40^, ^41^ This is an aspect of care which should be considered in the future. Figure 1 summarises the perceived benefits of ePROMs post‐RT.

Participants who received 15 or more treatments to their breast, felt that incorporating an ePROM during their treatment would also have been very useful. However, participants who received 5 or 10 treatments to their breast, generally did not feel as much of a need for an ePROM during RT treatment. This finding aligns to previous research published from the Fast Forward trial, which found that side effects peaked in the 2 weeks post‐RT with shorter RT treatment schedules.^42^ While this study is only a small scale pilot evaluation, this finding is worth investigating further to establish whether patients who receive longer RT schedules, and usually have more advanced disease, would benefit from the addition of an ePROM during their RT treatment as well as post‐RT.

Participants viewed it favourably when clinicians discussed their ePROM responses in the 12 week post‐RT appointment. In a study by Madsen et al.,^43^ patients with breast cancer described clinician preparedness as one of the most important aspects in building trust with their clinician. Consequently, clinicians should prioritise referring to the ePROM for information prior to speaking with each patient at their post‐RT appointment to aid trust building with their patients.

### Participants' perceptions of ease of use of the system and potential barriers to using the system

There were a number of significant aspects mentioned which prevented engagement with the ePROM. The most significant issue arising was the inability of participants to recognise and/or open the ePROM link. Through subsequent investigation, it was discovered that the link expired daily if not accessed on the same day. To resolve this issue, information regarding link expiration time should be explored and included in ePROM educational material provided to the patient and highlighted to patients during education.

Another common reason for not completing the ePROM was concern regarding confidentiality and/or apprehension to open a link from an unknown sender. To address this issue, ePROM suppliers should ensure inclusion of the RT department name into the subject line of the email and/ or amend the body of the email to include hospital specific information. Given that scam e‐mails have been determined to account for >70% of all e‐mails sent,^44^ participation is likely to be positively impacted by clearly identifiable e‐mail addresses, names and titles.

A small number of participants described forgetting to complete the ePROM or having insufficiently absorbed information regarding the ePROM completion process. With almost half of the participants in the study having also completed chemotherapy, this group is vulnerable to fatigue and ‘chemo brain’ symptoms including impact to memory, attention and processing speed.^45^ Some patients also present for RT with high levels of anxiety.^14^, ^39^ All of these factors can impact retention of information, emphasising the importance of explaining ePROM information to patients more than once; 1 week prior to the end of their RT treatment and again on their last day of RT.

No association was found between participants' ability to access the ePROM and individual characteristics including employment status, age and residential location. Previous publications which have found lower access to digital services like email based on patients' increasing age found that people over 75 years of age are most impacted.^46^, ^47^ Given that few participants in this study were over 75 years old, future study of this participant age group would be useful and add to these findings. While globally internet access in more rural areas has been a barrier to healthcare,^48^ no differences in internet access were noted between participants living in highly populated areas compared to those in lower populated areas.

An association was found between education level and ability to access the ePROM with those with higher levels of education accessing the ePROM most frequently. Health literacy is frequently demonstrated to be correlated to education level^49^ and lower health literacy can negatively impact access to ePROMs.^50^ Computer literacy is also an aspect which increases with higher education level.^51^ This findings further emphasises the need for allocating sufficient time with patients during ePROM information sessions to ensure that patients feel confident in understanding both how to access the ePROM and what is being asked in the ePROM.

## Strengths and limitations

Given that the average patient survey response rate is between 35 and 50%,^29^, ^52^ it can be concluded that the response rate (52%), shows acceptable engagement with and delivery of this pilot service evaluation. However, there are limitations of this study which should be addressed. One limitation is that the ePROM was only recently implemented in this single centre and therefore teething issues related to the technology, hampered data collection. Additionally, a limited number of participants reported significant side‐effects and therefore it is difficult to accurately establish the efficiency of this system. Very few participants reported that they utilised other services while receiving RT, so it is difficult to draw conclusions on their perception of access to Allied Health Professionals for services like wound dressing and emotional support.

## Conclusion

This pilot study demonstrated that incorporation of the ePROM into post‐RT care is a feasible option and can enhance care after breast irradiation. Post‐RT ePROMs have the potential to offer patients rapid access to care and optimise the distribution of follow‐up appointments for patients based on their individually assessed needs. Breast irradiation schedules vary considerably so the timing of ePROMs post‐RT should be optimised to capture and manage side‐effects for each patient as side‐effects arise in real time. Post‐RT, ePROMs offer reassurance and support to patients while waiting for their first post‐RT follow‐up appointment. Consideration should be given to offering access to ePROMs during RT to patients with longer RT schedules. ePROMs during and after RT treatment could help the management of not only physical side‐effects, but also patients' psychological wellbeing.

This study highlights the logistics/communication issues which can arise with administration of ePROMs post‐RT where patients are no longer seeing healthcare professionals for guidance. Consistent with previous findings,^53^ this study demonstrated that in the early stages of implementation, refinement of the ePROM process may be necessary through frequent service evaluation to ensure that the needs of the targeted population are being met.

While the feasibility of incorporating ePROMs after cancer treatment has recently been explored in other studies,^38^, ^53^, ^54^, ^55^ few publications have explored the use of ePROMs with patients post‐RT for breast cancer. Larger multicentre studies are needed to fully understand the potential benefits of ePROMs post‐RT for breast RT and other RT treatment sites. Such multicentre studies could be used for predicting future outcomes and the impact of this system on resources^21^ for a range of cancer sites.

## Conflict of Interest

No conflicts of interest exist for this study. No funding was provided by an external company for this research. However, the ePROM software, was supplied as part of an ongoing departmental contract with Cievert at no additional charge and in line with local change control processes.

## Ethical Approval

Ethical approval was obtained through Ulster University and the Western Health and Social Care Trust (WHSCT), Northern Ireland.

## Supporting information


**Appendix S1.** Combined terminology criteria for reporting of adverse effects.

**Appendix S2.** Evaluation survey.

## Data Availability

The data that support the findings of this study are available on request from the corresponding author. The data are not publicly available due to privacy or ethical restrictions.

## References

[1] Breast Cancer Treatment. Available from: https://www.cancerresearchuk.org/health‐professional/cancer‐statistics/statistics‐by‐cancer‐type/breast‐cancer#heading‐Six (accessed 8 May 2023).

[2] Lewis P , Brunt AM , Coles C , Griffin S , Locke I , Roques T . Moving forward fast with FAST‐Forward. Clin Oncol 2021; 33: 427–429. 10.1016/j.clon.2021.04.007 33994270

[3] Hille‐Betz U , Vaske B , Bremer M , et al. Late radiation side effects, cosmetic outcomes and pain in breast cancer patients after breast‐conserving surgery and three‐dimensional conformal radiotherapy. Strahlenther Onkol 2016 Jan; 192: 8–16. 26416291 10.1007/s00066-015-0899-y

[4] The Institute of Cancer Research . One‐week course of radiotherapy could benefit women with early stage breast cancer, study finds. 2020. Available from: https://www.icr.ac.uk/news‐archive/one‐week‐course‐of‐radiotherapy‐could‐benefit‐women‐with‐early‐stage‐breast‐cancer‐study‐finds (accessed 12 May 2023).

[5] Dilalla V , Chaput G , Williams T , Sultanem K . Radiotherapy side effects: integrating a survivorship clinical lens to better serve patients. Curr Oncol 2020; 27: 107–112. 32489253 10.3747/co.27.6233 PMC7253739

[6] Royal College of Radiographers (RCR) . Postoperative radiotherapy for breast cancer: UK consensus statements. 2019. Available from: https://www.rcr.ac.uk/publication/postoperative‐radiotherapy‐breast‐cancer‐uk‐consensus‐statements (accessed 12 May 2023).

[7] Royal College of Radiographers (RCR) . Timely delivery of radical radiotherapy: Guidelines for the management of unscheduled treatment interruptions. 2019. Available from: https://www.rcr.ac.uk/publication/timely‐delivery‐radical‐radiotherapy‐guidelines‐management‐unscheduled‐treatment (accessed 12 May 2023).

[8] Royal College of Radiographers (RCR) . Radiotherapy dose fractionation. 2019. Available from: https://www.rcr.ac.uk/publication/radiotherapy‐dose‐fractionation‐third‐edition (accessed 12 May 2023).

[9] National Institute of Health Care Excellence (NICE) . Early and locally advanced breast cancer: diagnosis and management. Evidence reviews for breast radiotherapy. 2018. Available from: https://www.nice.org.uk/guidance/ng101/evidence (accessed 12 May 2023).

[10] Lafranconi A , Pylkkänen L , Deandrea S , et al. Intensive follow‐up for women with breast cancer: review of clinical, economic and patient's preference domains through evidence to decision framework. Health Qual Life Outcomes 2017; 15: 1–8. 29052503 10.1186/s12955-017-0779-5 PMC5649085

[11] NHS Digital . 2022. Available from: https://digital.nhs.uk/data‐and‐information/data‐tools‐and‐services/data‐services/patient‐reported‐outcome‐measures‐proms/background‐information‐about‐proms (accessed 12 May 2023).

[12] Palmen LN , Schrier JC , Scholten R , Jansen JH , Koëter S . Is it too early to move to full electronic PROM data collection?: a randomized controlled trial comparing PROM's after hallux valgus captured by e‐mail, traditional mail and telephone. Foot Ankle Surg 2016; 22: 46–49. 26869500 10.1016/j.fas.2015.05.001

[13] Gabbard J , McLouth CJ , Brenes G , et al. Rapid electronic capturing of patient‐reported outcome measures in older adults with end‐stage renal disease: a feasibility study. Am J Hosp Palliat Med 2021; 38: 432–440. 10.1177/1049909120954805 PMC8216503 32935548

[14] Halkett G , O'Connor M , Aranda S , et al. Communication skills training for radiation therapists: preparing patients for radiation therapy. J Med Radiat Sci 2016; 63: 232–241. 27741388 10.1002/jmrs.171 PMC5167288

[15] Stover A , Irwin DE , Chen RC , et al. Integrating patient‐reported outcome measures into routine cancer care: cancer patients' and clinicians' perceptions of acceptability and value. Egems 2015; 3(1): 1–22. 10.13063/2327-9214.1169 PMC4636110 26557724

[16] Hu X , Zhang C , Zhang Y . BC‐PROM: validation of a patient‐reported outcomes measure for patients with breast cancer. Medicine (Baltimore) 2017; 96: e6781. 28445314 10.1097/MD.0000000000006781 PMC5413279

[17] National Institute of Health Care Excellence (NICE) . Patient experience in adult NHS services: improving the experience of care for people using adult NHS services. 2021. Available from: https://www.nice.org.uk/guidance/cg138 (accessed 12 May 2023). 34260162

[18] Donaldson L , Rutter P , Henderson M . The right time, the right place. 2014. Available from: https://www.health‐ni.gov.uk/publications/right‐time‐right‐place (accessed 12 May 2023).

[19] Benoga R . Systems, not structures: changing health and social care. 2016. Available from: https://www.health‐ni.gov.uk/sites/default/files/publications/health/expert‐panel‐full‐report.pdf (accessed 12 May 2023).

[20] O'Poustie M . Health & well being 2026 – delivering together. 2017. Available from: https://www.health‐ni.gov.uk/sites/default/files/publications/health/health‐and‐wellbeing‐2026‐delivering‐together.pdf (accessed 12 May 2023).

[21] Ishaque S , Karnon J , Chen J , Nair R , Salter AB . A systematic review of randomised controlled trials evaluating the use of patient‐reported outcome measures. Qual Life Res 2018; 28: 567–592. 30284183 10.1007/s11136-018-2016-z

[22] National Health Service (NHS) . The NHS long term plan. 2019. Available from: https://www.longtermplan.nhs.uk/online‐version/overview‐and‐summary/ (accessed 12 May 2023).

[23] Polgar S , Thomas SA . Introduction to Research in the Health Sciences, 6th edn. Churchill Livingstone Press, Edinburgh, 2013. Chapter 1, 3 and 4.

[24] Lim E , Baldwin D , Beckles M , et al. Guidelines on the radical management of patients with lung cancer. Thorax 2010; 65(Suppl 3): iii1–iii27. 20940263 10.1136/thx.2010.145938

[25] Rattay T , Seibold P , Aguado‐Barrera ME , et al. External validation of a predictive model for acute skin radiation toxicity in the REQUITE breast cohort. Front Oncol 2020; 10: 575909. 33216838 10.3389/fonc.2020.575909 PMC7664984

[26] Cancer Focus (Northern Ireland) . Breast Cancer. Available from: https://cancerfocusni.org/cancer‐info/types‐of‐cancer/signs‐of‐breast‐cancer‐be‐breast‐aware/ (accessed 12 May 2023).

[27] Thabane L , Ma J , Chu R , et al. A tutorial on pilot studies: the what, why and how. BMC Med Res Methodol 2010; 10: 1–10. 20053272 10.1186/1471-2288-10-1 PMC2824145

[28] Schober P , Bossers SM , Dong PV , Boer C , Schwarte LA . What do anesthesiologists know about p values, confidence intervals, and correlations: a pilot survey. Anesthesiol Res Pract 2017; 12: 2015–2017. 10.1155/2017/4201289 PMC5660771 29158732

[29] Raosoft . Sample size calculator. 2004. Available from: http://www.raosoft.com/samplesize.html (accessed 12 May 2023).

[30] Perneger TV , Peytremann‐Bridevaux I , Combescure C . Patient satisfaction and survey response in 717 hospital surveys in Switzerland: a cross‐sectional study. BMC Health Serv Res 2020; 20: 1–8. 10.1186/s12913-020-5012-2 PMC7052977 32122346

[31] Mishra P , Pandey CM , Singh U , Gupta A , Sahu C , Keshri A . Descriptive statistics and normality tests for statistical data. Ann Card Anaesth 2019; 22: 67–72. 30648682 10.4103/aca.ACA_157_18 PMC6350423

[32] Orcan F . Parametric or non‐parametric: Skewness to test normality for mean comparison. Int J Assess Tools Educ 2020; 7: 255–265.

[33] Neideen T , Brasel K . Understanding statistical tests. J Surg Educ 2007; 64: 93–96. 17462209 10.1016/j.jsurg.2007.02.001

[34] McHugh ML . The chi‐square test of independence. Biochem Med 2013; 23: 143–149. 10.11613/BM.2013.018 PMC3900058 23894860

[35] Williams M , Moser T . The art of coding and thematic exploration in qualitative research. Int Manage Rev 2019; 15: 45–55.

[36] Meyrick J . What is good qualitative research? A first step towards a comprehensive approach to judging rigour/quality. J Health Psychol 2006; 11: 799–808. 16908474 10.1177/1359105306066643

[37] Basch E , Abernethy AP , Mullins CD , et al. Recommendations for incorporating patient‐reported outcomes into clinical comparative effectiveness research in adult oncology. J Clin Oncol 2012; 30: 4249–4255. 23071244 10.1200/JCO.2012.42.5967

[38] Hauth F , Bizu V , App R , et al. Electronic patient‐reported outcome measures in radiation oncology: initial experience after workflow implementation. JMIR Mhealth Uhealth 2019; 7: e12345. 31342906 10.2196/12345 PMC6685133

[39] Halkett GK , Kristjanson LJ , Lobb E , et al. Information needs and preferences of women as they proceed through radiotherapy for breast cancer. Patient Educ Couns 2012; 86: 396–404. 21664788 10.1016/j.pec.2011.05.010

[40] Licht T , Nickels A , Riedl D , Rumpold G , Holzner B . Evaluation of inpatient cancer rehabilitation by routine electronic patient‐reported outcome measures (ePROM): Improvement of quality of life (QoL) and psychological distress. J Clin Oncol 2019; 37(15_suppl): e18294.

[41] Dempsey K , Saw R , Bartula I , et al. Embedding electronic patient‐reported outcome measures into routine care for patients with stage III MELanoma (ePROMs‐MEL): protocol for a prospective, longitudinal, mixed‐methods pilot study. BMJ Open 2022; 12: e066852. 10.1136/bmjopen-2022-066852 PMC9772660 36600423

[42] Brunt AM , Wheatley D , Yarnold J , et al. Acute skin toxicity associated with a 1‐week schedule of whole breast radiotherapy compared with a standard 3‐week regimen delivered in the UK FAST‐Forward Trial. Radiother Oncol 2016; 120: 114–118. 27046390 10.1016/j.radonc.2016.02.027 PMC4998960

[43] Madsen SM , Holm S , Riis P . Participating in a cancer clinical trial? The balancing of options in the loneliness of autonomy: a grounded theory interview study. Acta Oncol 2007; 46: 49–59. 17438705 10.1080/02841860600911164

[44] Datar TD , Cole KA , Rogers MK . Awareness of scam e‐mails: an exploratory research study. Annual ADFSL Conference on Digital Forensics, Security and Law, 2014; Vol 12, 10–34. Available from: https://commons.erau.edu/adfsl/2014/wednesday/12

[45] Kovalchuk A , Kolb B . Chemo brain: from discerning mechanisms to lifting the brain fog—an aging connection. Cell Cycle 2017; 16: 1345. 28657421 10.1080/15384101.2017.1334022 PMC5539816

[46] Meirte J , Hellemans N , Anthonissen M , et al. Benefits and disadvantages of electronic patient‐reported outcome measures: systematic review. JMIR Perioperat Med 2020; 3: e15588. 10.2196/15588 PMC7709853 33393920

[47] Schamber EM , Takemoto SK , Chenok KE , Bozic KJ . Barriers to completion of patient reported outcome measures. J Arthroplasty 2013; 28: 1449–1453. 23890831 10.1016/j.arth.2013.06.025

[48] Padala KP , Wilson KB , Gauss CH , Stovall JD , Padala PR . VA video connect for clinical care in older adults in a rural state during the COVID‐19 pandemic: cross‐sectional study. J Med Internet Res 2020; 22: e21561. 32936773 10.2196/21561 PMC7537724

[49] Jansen T , Rademakers J , Waverijn G , Verheij R , Osborne R , Heijmans M . The role of health literacy in explaining the association between educational attainment and the use of out‐of‐hours primary care services in chronically ill people: a survey study. BMC Health Serv Res 2018; 18: 1–3. 29855365 10.1186/s12913-018-3197-4 PMC5984471

[50] Long C , Beres LK , Wu AW , Giladi AM . Patient‐level barriers and facilitators to completion of patient‐reported outcomes measures. Qual Life Res 2021; 31: 1–8. 34533759 10.1007/s11136-021-02999-8

[51] Chang SL , Shieh RS , Liu EZ , Yu PT . Factors influencing women's attitudes towards computers in a computer literacy training program. Turk Online J Educat Technol 2012; 11: 177–187.

[52] NHS . GP patient survey 2021. 2021. https://www.england.nhs.uk/statistics/2021/07/08/gp‐patient‐survey‐2021/ (accessed 12 May 2023).

[53] Schuler T , Hruby G , Wong S , Grimberg K , Kneebone A , Eade T . Patient‐reported outcome measures during routine care palliative radiotherapy: implementation experience and completion rates. *European Congress of Radiology‐2021 ASM* .

[54] Crockett C , Price J , Pham M , et al. Experience with the routine use of electronic patient‐reported outcome measures for patients with lung cancer. JCO Clin Cancer Inform 2023; 7: e2200150. 37071029 10.1200/CCI.22.00150 PMC10281443

[55] Pantiora E , Hedman L , Sjökvist O , Karakatsanis A . Electronic Patient Reported Outcome Measures (ePROMs) in patients with breast disease: an open‐label, randomized controlled trial on patient reported experience measures (PREMs) and effectivity. Eur J Surg Oncol 2022; 48: e33.

