# Supplementary material for: Patients' experiences of engaging with electronic Patient Reported Outcome Measures (PROMs) after the completion of radiation therapy for breast cancer: a pilot service evaluation
Source: J Med Radiat Sci. 2023 Aug 7;70(4):424–35. doi: 10.1002/jmrs.711 (PMC10715367; doi:10.1002/jmrs.711)
Supplement: Supplementary file 1 — Appendix S1. Combined terminology criteria for reporting of adverse effects. [file JMRS-70-424-s002.docx]

**Appendix 1: Combined Terminology Criteria for reporting of adverse effects**

Under each of the symptoms, please select the option that is most appropriate- Thank you.

**Symptom: Skin changes**

1: No change to skin

2: Mild redness or dry flaking skin

3: Bright redness; Skin tight and sore; Local skin peeling with some clear ooze

4: Bright redness; Skin tight and sore; Large areas of skin peeling with some clear ooze

5: Bright redness; Skin tight and sore; Large areas of skin peeling with a lot of clear ooze

**Symptom: Itch**

1: None

2: Mild or local itching

3: Intense or widespread itch, responding to cream or oral medication

4: Intense or widespread itch, not responding to cream or oral medication

**Symptom: Breast swelling**

1: None

2: Mild swelling

3: Moderate swelling

4: Severe swelling

5: Severe swelling with ulceration

**Symptom: Fatigue**

1: None

2: Mild-doses not affect you normal

3: Moderate: Affects some of your activities

4: Severe: Unable to carry out normal activities without help

5: Bed bound: Unable to get out of bed

**Symptom: Breast pain**

1: None

2: Mild pain

3: Moderate pain. Pain relief is required

4: Severe pain you need to take pain relief and it affects your normal activities

5: Pain not responding to pain relief, unable to carry out normal activities

If a highlighted answer is selected, your clinician will contact you within 2 days, from a withheld number. If you are not contacted, please follow standard procedure using the phone numbers supplied.
