# Supplementary material for: Patients' experiences of engaging with electronic Patient Reported Outcome Measures (PROMs) after the completion of radiation therapy for breast cancer: a pilot service evaluation
Source: J Med Radiat Sci. 2023 Aug 7;70(4):424–35. doi: 10.1002/jmrs.711 (PMC10715367; doi:10.1002/jmrs.711)
Supplement: Supplementary file 2 — Appendix S2. Evaluation survey. [file JMRS-70-424-s001.docx]

**Appendix 2: Evaluation Survey**

Please tick the appropriate box provided:

**SECTION A**

Part 1: About you:

1. What is your current employment status?

| Employed but on leave |  |
| --- | --- |
| Working part-time |  |
| Working full time |  |
| Retired |  |
| Other | Please state _________________________ |

1. Which of the following best describes your educational level?

| Primary school |  |
| --- | --- |
| Secondary school |  |
| Diploma |  |
| Undergraduate degree |  |
| Postgraduate degree |  |
| Other | Please state _______________________ |
|  |  |

1. What is your age?

| 18-25 years |  |
| --- | --- |
| 26-35 years |  |
| 36-45 years |  |
| 46-55 years |  |
| 56-65 years |  |
| 66-75 years |  |
| 76-85 years |  |
| + 85 years |  |

1. What type of location do you live in?

| City |  |
| --- | --- |
| Town |  |
| Village |  |
| Countryside |  |
| Remote |  |
|  |  |

Part 2: About your treatment at NWCC:

1. How many radiotherapy treatments did you receive in total?

2. Did you have any other treatments, before your radiotherapy started?

(Tick all that apply)

| Surgery |  |
| --- | --- |
| Chemotherapy |  |
| Hormones |  |
| Other | Please state _______________________ |
|  |  |

3. Did you require any of the following, while receiving radiotherapy?

(Tick all that apply)

| Wound dressing |  |
| --- | --- |
| Seroma drainage |  |
| Emotional support |  |
| Other | Please state _______________________ |

______________________________________________________________

____________________________________________________________________________________________________________________________

4. Did you visit any other allied health professionals while receiving radiotherapy? (Tick all that apply)

| Physiotherapist |  |
| --- | --- |
| Speech & Language therapist |  |
| Occupational Health therapist |  |
| Dietitian |  |
| Hospital Social Worker |  |
| Other | Please state ________________ |

______________________________________________________________

____________________________________________________________________________________________________________________________

Part 3: About the ePROM:

1. Were you able to access the ePROM questionnaire?

| Yes- continue with survey |  |
| --- | --- |
| No- please say why then continue to section B |  |

If you answered ‘No’, please provide more details:

______________________________________________________________

____________________________________________________________________________________________________________________________

1. Did you complete the ePROM questionnaire?

| Yes- continue with survey |  |
| --- | --- |
| No- please say why then continue to section B |  |

If you answered ‘No’, please provide more details:

______________________________________________________________

____________________________________________________________________________________________________________________________

1. Did you use the ePROM to report that you were experiencing side-effects that required a response?

| Yes- continue with survey |  |
| --- | --- |
| No- continue to question 7 |  |

1. If you reported side-effects which required a response, were you then contacted by a member of the radiotherapy clinical team?

| Yes- continue with survey |  |
| --- | --- |
| No- used the triage helpline instead |  |
| No- waited until scheduled follow up appointment |  |
| Other- Please state ___________________________ |  |
|  |  |

1. If contacted, how long did it take to get a response?

_________________________________________________

Please add any additional comments here about the timing of this contact:

______________________________________________________________

____________________________________________________________________________________________________________________________

1. Overall, how would you rate the quality of care that you received through completion of the ePROM?

| Very poor |  |
| --- | --- |
| Poor |  |
| Adequate |  |
| Good |  |
| Very good |  |

Please add any additional comments here about the care that you received based on completion of the ePROM:

______________________________________________________________

____________________________________________________________________________________________________________________________

1. At your 12 week follow up appointment, the clinician will have reviewed your ePROM questionnaire submission from six weeks after your Radiotherapy finished. Did you feel that this aided the review process?

| Yes |  |
| --- | --- |
| No |  |

Please provide relevant details:

______________________________________________________________

____________________________________________________________________________________________________________________________

**SECTION B**

1. Did you feel that the ePROM system was explained sufficiently?

| Yes |  |
| --- | --- |
| No |  |

If you answered ‘No’, please provide more details:

______________________________________________________________

____________________________________________________________________________________________________________________________

1. Were you apprehensive to use the ePROM system?

| Yes |  |
| --- | --- |
| No |  |

If you answered ‘Yes’, please provide more details:

______________________________________________________________

____________________________________________________________________________________________________________________________

1. Would you recommend using the ePROM system to report side effects after your radiotherapy treatment has finished?

| Yes |  |
| --- | --- |
| No |  |

If you answered ‘No’, please provide more details:

______________________________________________________________

____________________________________________________________________________________________________________________________

1. Do you think that ePROM questionnaires should be sent whilst receiving radiotherapy to report side effects?

| Yes |  |
| --- | --- |
| No |  |

If you answered ‘No’, please provide more details:

______________________________________________________________

____________________________________________________________________________________________________________________________

1. Do you feel the information and support you received has helped you to manage your post-radiotherapy care?

| Yes |  |
| --- | --- |
| No |  |

Please provide more details:

______________________________________________________________

____________________________________________________________________________________________________________________________

1. Please let us know if you have any suggestions on how to improve the use of the ePROM system for reporting radiotherapy related issues:

______________________________________________________________

____________________________________________________________________________________________________________________________

______________________________________________________________

______________________________________________________________

**Thank you for taking the time to complete this survey.**

**Your feedback is invaluable and much appreciated.**
